# *Festulolium* and fungal endophyte associations: host status for *Meloidogyne incognita* and nematotoxic plant extracts

**DOI:** 10.21307/jofnem-2020-076

**Published:** 2020-07-28

**Authors:** Susan L. F. Meyer, Brian J. Patchett, Timothy J. Gillanders, Mihail R. Kantor, Patricia Timper, Margaret H. MacDonald

**Affiliations:** 1 USDA ARS Mycology and Nematology Genetic Diversity and Biology Laboratory, Northeast Area, Henry A. Wallace Beltsville Agricultural Research Center (BARC)-West, Beltsville, MD, 20705; 2 Cropmark Seeds Ltd, 49 Manion Road, RD7 Christchurch, 7677, New Zealand; 3 USDA ARS Crop Protection and Management Research Unit, Southeast Area, Tifton, GA 31793

**Keywords:** *Epichloë*, *Festuca*, *Festulolium*, Fungal endophyte, Interaction, *Lolium*, *Meloidogyne incognita*, Nematode host status, Plant extract, Root-knot nematode

## Abstract

*Festulolium* hybrids are forage grasses used worldwide in temperate climates. They are associated with the fungal endophyte *Epichloë uncinata*, which aids in nutrient uptake, drought tolerance, and production of metabolites that protect against parasites and herbivores. *Epichloë uncinata* produces loline alkaloids, which can deter insect pests. *Festulolium* has not been widely studied for susceptibility to plant-parasitic nematodes, so *Festulolium* lines, with and without fungal endophytes, were tested in the greenhouse for host status to the root-knot nematode *Meloidogyne incognita*. All were poor hosts, regardless of line or endophyte status. Pepper seedlings planted into soil following removal of the *Festulolium* plants were infected by nematodes, likely because of surviving nematodes from the original inoculation combined with some reproduction on *Festulolium*. Lolines were found in shoots and roots of all endophyte-associated lines, and some types of lolines in roots increased after nematode infection. Methanolic extracts from roots and shoots of a tested *Festulolium* line did not inhibit egg hatch, but killed nearly a third of second-stage juveniles whether an endophyte was present or not. Further studies would indicate whether these *Festulolium* lines aid in suppressing field populations of *M. incognita*.

Cool-season grasses (family Poaceae) are often associated with claviceptaceous endophytic fungi in the genus *Epichloë* (Schardl et al., 2004; König et al., 2018). The hyphae of these fungi grow via intercalary hyphal extension from the basal meristem of the host plant, eventually colonizing the new seed when the host reaches maturity (Christensen et al., 2008). The grass benefits from this association in multiple ways, including increased uptake of nutrients, improved vigor during drought, and production of metabolites that protect against parasites and herbivores (Schardl et al., 2004; Schouten, 2016). However, the endophytes can produce indole-diterpene alkaloids and ergot alkaloids in pasture grasses, resulting in toxicity to livestock (Schardl et al., 2004). Consequently, associations have been developed with fungal endophytes that produce little or no ergot alkaloids or the indole-diterpene alkaloid lolitrem B (Timper and Bouton, 2012; Young et al., 2013; Fletcher et al., 2017).

One such association occurs with the fungal endophyte *Epichloë uncinata* (W. Gams, Petrini and D. Schmidt) and *Festulolium* hybrids, which are important forage grasses used for pastoral agriculture in temperate climates across the world. The *Festulolium* hybrids are intergeneric crosses between *Festuca pratensis* (Huds.) and *Lolium perenne* (L.) and/or *L. mulitflorum* (Lam.). *Festuca pratensis* is the natural grass host of *E. uncinata*, an asexual species and interspecific hybrid likely descended from a hybridization event between the sexual species *E. typhina* and *E. bromicola* (Schardl et al., 2012; Saikkonen et al., 2016). Wild populations of *F. pratensis* are often found to be infected with *E. uncinata* at a high frequency (Cagnano et al., 2019), suggesting a strong mutualistic relationship with benefits to both host and endophyte.

*Epichloë uncinata* produces bioprotective loline alkaloids, which can accumulate to 2% of the host plant dry weight (Zhang et al., 2009). The loline alkaloids are water soluble and able to translocate around host tissues to areas such as the roots, where the endophyte itself is not found actively growing (Patchett et al., 2008). Importantly, loline alkaloids do not cause the animal health disorders (fescue toxicosis and ryegrass staggers) in grazing livestock associated with some of the other endophyte-produced alkaloids, such as ergovaline and lolitrem B (Gooneratne et al., 2012; Fletcher et al., 2017). The biocontrol benefits that loline alkaloids provide to pastoral agriculture farming systems have led to the commercialization of many loline-producing endophyte strains of *E. uncinata*, including the U2 endophyte strain used in the current study. In contrast to lack of toxicity to grazing mammals, the loline alkaloids produced by *E. uncinata* may be a feeding deterrent, or toxic, to a wide range of insect pests (Riedell et al., 1991; Matsukura et al., 2012; Barker et al., 2015a, b; Nboyine et al., 2017). However, studies with plant-parasitic nematodes and lolines indicated that the loline alkaloid N-formylloline could either attract or repel the plant-parasitic nematode *Pratylenchus scribneri*, depending on the loline concentration (Bacetty, Snook, Glenn, Noe, Nagabhyru and Bacon, 2009).

While endophytes can affect susceptibility of grasses to nematodes, host status may be more strongly influenced by plant cultivar than by presence or absence of endophyte. For example, tall fescue ‘Kentucky 31’, with or without endophytes, was a host for the Southern root-knot nematode *Meloidogyne incognita* (Kofoid and White) Chitwood (Jia et al., 2013). Similarly, host status of tall fescue ‘Jesup’ to *M. incognita* did not depend on fungal colonization; the cultivar was a nonhost regardless of endophyte status (Nyczepir and Meyer, 2010).

As indicated by the examples given above, interactions among grasses, endophytes, and nematodes are complex. The current study was conducted to investigate host status of five *Festulolium* lines, each with and without its own strain of loline-producing *E. uncinata* endophyte, to *M. incognita. Meloidogyne incognita* was selected for this study because it is an economically important species that attacks many plant hosts (Jones et al., 2013). Consequently, suppression of this spp. would be beneficial for the grasses and for other plants in the same fields (Jia et al., 2013; Nyczepir et al., 2014). The goal was to determine whether nematode reproduction varied with *Festulolium* line, presence of endophyte, or both. To compare *M. incognita* populations on a crop following the different *Festulolium* lines, susceptible pepper seedlings were transplanted into the soil from which inoculated *Festulolium* plants had been harvested. Additionally, methanolic extracts from roots and shoots of a *Festulolium* line, plus and minus endophyte, were assayed for production of metabolites active against *M. incognita*.

## Materials and methods

### *Festulolium* lines and endophytes

Five *Festulolium* hybrids were used for this study (Table 1). Each hybrid contained different degrees of *F. pratensis*, *L. perenne*, and *L. multiflorum* genetics integrated through at least six years of conventional plant breeding (Appendix 1). Each hybrid was associated with a different strain of *E. uncinata* originating from four different geographical origins: U2 (Norway), U5 (Germany), U6 (Bulgaria), and U8 and U10 (Sweden). To obtain nil controls (E−) of each hybrid, seed was placed in a humidity chamber (Contherm Phytotron Climate Simulator, Wellington, New Zealand) at 45°C/50% R.H. for 21 d to remove the endophyte infection. Endophyte infection status of individual plants grown for experiments was confirmed via histological staining of the inner leaf sheath with aniline blue, followed by visualization with a microscope (Clark et al., 1983) or by use of a commercial endophyte tiller test kit (*Epichloë* Endophyte Tissue Print Immunoblot Tiller Kit; Cropmark Seeds Ltd., Christchurch, New Zealand). Plants with and without endophyte are designated E+ and E−, respectively.

**Table 1.** *Festulolium* lines and *Epichloë uncinata* endophytes used in the experiments.

*Festulolium* genotype and endophyte number	*Epichloë uncinata* strain^a^
FHCF0802	U2 E− (Endophyte-free)
FHCF0802 2348M	U2 E+
FHAC0802	U5 E− (Endophyte-free)
FHAC0802 ABA 10-23	U5 E+
FHCD0802	U6 E− (Endophyte-free)
FHCD0802 BUS 10-12	U6 E+
FHAB0802	U8 E− (Endophyte-free)
FHAB0802 ABA 10-22	U8 E+
FHCD0802	U10 E− (Endophyte-free)
FHCD0802 BUS 10-13	U10 E+

**Note:** aIn the text of this paper, the *Festulolium*/endophyte associations are generally referred to by the *E. uncinata* strain. For example, FHCF0802 as U2 E−, and FHCF0802 2348M as U2 E+.

### Nematode cultures

*Meloidogyne incognita* race 1 was obtained as described in Meyer et al. (2016). Susceptible cayenne pepper (*Capsicum annuum*) ‘PA-136’ plants were inoculated with *M. incognita* (originally isolated in Maryland), and maintained in a greenhouse (24-29°C; natural and supplemental lighting combined for a 16-hr daylength). All greenhouse experiments described in this paper were conducted at this location under the same conditions.

Egg masses were picked from roots of pepper plants 2 to 3 mon after inoculation, and eggs were separated in 0.6% sodium hypochlorite for 5 min followed by a sterile distilled water (SDW) rinse. For microwell assays with second-stage juveniles (J2) directly immersed in extracts, sterilized eggs were placed into a hatching chamber (Spectra/Mesh Nylon Filter, openings 25-µm-diam.; Spectrum Laboratories Inc., Rancho Dominguez, CA) in an autoclaved dish, and placed on a rotary shaker (35 rpm for 3 d).

### Greenhouse experiments

All *Festulolium* lines, with and without endophytes, were grown in steamed, dried 16:9 sand/soil mix (82.6% sand, 9.5% silt, and 8.0% clay) classified as loamy sand. At planting, 15-cm-diam. pots were each sown with several seeds, with one type of *Festulolium*/endophyte association per pot. *Festulolium* seedlings were first tested at about 6 wk for the presence of the endophyte as indicated above, and then thinned to one plant per pot. For host status trials, susceptible pepper ‘PA-136’ was planted in Promix PGX (Premier Tech Horticulture, Quakertown, PA) about 1 wk after planting fescue as a positive control to indicate that the nematode inoculum was viable. At 4 to 8 wk (depending on *Festulolium* and endophyte development) after *Festulolium* planting, the pepper seedlings (5-7 wk old) were transplanted into soil in pots. Inoculated plants for host status tests each received an aqueous suspension (ca. 16,000 eggs total in 5 ml water) containing eggs at various developmental stages, including 5,000 eggs with either a J1 (first-stage juvenile) or J2. This was inoculated into several holes in the soil near the base of each plant. Treatments in tests for host status of *Festulolium* endophytes to *M. incognita* were: (i) inoculated fallow soil (six pots), pepper (six pots), and U2 E± and U5± (three pots of each) in each of two trials, and (ii) inoculated fallow soil (six pots), pepper (six pots), and U6±, U8±, and U10± (three pots of each) in each of two other trials. Pots were arranged in a randomized complete block design. *Festulolium* and pepper plants were harvested 5 to 6 wk after inoculation, and shoots were weighed, roots were rinsed and root fresh weights recorded, gall indices assigned, and eggs collected and counted from inoculated plants. Egg collecting and counting procedures (to estimate the number per root system and per g of root) were as follows: roots were cut, blended in 0.6% sodium hypochlorite for 1 min at low speed, and rinsed with water. The resulting egg suspension was poured through no. 60 over no.230 nested sieves (pore sizes 250 and 63-µm diam.), collected on a no.500 sieve (pore size 25-µm diam.), resuspended in tap water (40 ml), diluted and counted. Gall indices follow Daulton and Nusbaum (1961). 0 = no galls, 1 = 1 to 4 galls, 5 = 5 to 25 galls, 10 = 26 to 100 galls, and 25 = more than 100 galls.

Soil was saved and mixed in the original pots, and a pepper seedling (at least 5 wk old) was transplanted into each pot that had contained inoculated *Festulolium*, pepper, or fallow soil. These pepper plants were harvested 5 to 6 wk after transplant. Root weights, gall indices, and egg counts were recorded. *Festulolium* plants used for nematode host status were destructively sampled and not used for loline analysis.

### Loline analysis of *Festulolium* root and shoot samples

Roots and shoots from all five *Festulolium* lines, ± endophyte, were analyzed for lolines from greenhouse-grown plants that had not been inoculated with *M. incognita* (greenhouse methods described above). Loline analysis was also conducted with roots and shoots from lines FHCF0802 2348M (U2 E+), FHCF0802 (U2 E−), FHAC0802 ABA10-23 (U5 E+), and FHAC0802 (U5 E−) that had been inoculated with *M. incognita*. Plants were harvested 11 to 14 wk after planting; plants with *M. incognita* were harvested 5 wk after nematode inoculation. Loline values are reported from 5 to 12 plants per *Festulolium*/endophyte association. Not all plants were collected from the same greenhouse trials, so statistical comparisons were not conducted.

Lolines were analyzed using a method modified from Yates et al. (1990) and Blankenship et al. (2001). A 250 mg sample of ground, freeze-dried plant material was extracted in 5 ml of extraction solvent (95:5 dichloromethane: ethanol) along with 250 µl saturated sodium bicarbonate in a 6 ml glass vial on an orbital shaker at 200 rpm for 1 hr. The extraction solvent contained 60 µg/ml 4-Phenomorpholine (Sigma Aldrich^®^, Sydney, Australia) as an internal standard. Samples were then filtered using a cotton-plugged pasteur pipette and 1 ml of the filtrate transferred to a 2 ml gas chromatography (GC) vial for analysis within 24 hr. Gas chromatography was carried out using a Shimadzu GC-2010 equipped with a flame ionization detector and a ZB-5 (30 m × 0.25 mm × 0.25 µm) capillary column (Phenomonex^®^, Auckland, New Zealand). Hydrogen was used as a carrier gas at a flow rate of 6 ml per min. H_2_ and air flows at the detector were 40 and 400 ml per min, respectively. The oven was heated from 40°C to 320°C at a rate of 20°C per min and held there for 5 min. Samples were introduced via 1 µl split-less injections. Retention times were as follows: *N*-methylloline (5.9 min), 4-phenomorpholine (6.9 min), *N*-acetylnorloline (8.2 min), *N*-formylloline (8.4 min), and *N*-acetylloline (8.7 min). The GC was standardized using loline standards purified from Barrier U2^TM^ seed (Cropmark Seeds Ltd., Christchurch, New Zealand) and a *Festulolium* cultivar infected with *E. uncinata*, using the methods of Briggs et al. (2017). The limit of detection was 30 µg/g.

### *Festulolium* U6 E+ and U6 E− root and shoot extract preparation and microwell assays

Shoots and roots of 1 ½ mon-old *Festulolium* U6 E+ and U6 E− plants (not inoculated with *M. incognita*) were harvested, weighed and frozen at −80°C. This line was selected for testing because it produced the most abundant amount of tissue, ensuring enough for extract production. The plant tissues were freeze-dried (FreeZone 4.5 freeze dryer, Labconco Corporation., Kansas City, MO) and finely ground in a Cyclone Sample Mill (UDY Corporation, Fort Collins, CO) fitted with a 1-mm-diam. pore sieve. Tissue was then milled to a fine powder. Extracts were prepared by placing 3 g of powdered tissue in 30 ml of 100% methanol in a 125 ml covered flask, which was placed on a rotary shaker (VWR, Advanced Digital Shaker, Radnor, PA) at room temperature (25°C) for 20 hr at 100 rpm. The extracts were vacuum-filtered through Whatman No. 2 filter paper (Whatman, Clifton, NJ). The filtered solution was split into two pre-weighed 50 ml conical tubes and dried in a vacuum centrifuge (Centrivap Concentrator, Labconco Corporation, Kansas City, MO) at 40°C for ca. 16 hr. The dried extracts were weighed and suspended in 100% dimethyl sulfoxide (DMSO), 40 mg/ml. The extracts were heated to 60°C for up to 30 min and vortexed until completely dissolved, and then filtered through 0.45 µm (Nalgene, Rochester, NY) and 0.2 µm (Whatman, Clifton, NJ) syringe filters. Four dilutions were made from each extract. To prevent contamination by microbes, kanamycin monosulfate (Phytotech Lab, Shawnee Mission, KS) was also added to the extract solutions so that the final concentration would be 25 µg/µl in all wells (except the water control without the antibiotic).

Methanolic extracts were tested for activity against *M. incognita* eggs and J2 in 96-well polystyrene plates, following procedures in Meyer et al. (2006). Each treatment was placed in five replicate wells in each of two trials (10 wells total). Treatments in the wells were 400 µg/ml extract + 1% DMSO, 200 µg/ml extract + 0.5% DMSO, 100 µg/ml extract + 0.25% DMSO, and 50 µg/ml extract + 0.125%, corresponding DMSO controls, an SDW water control, and an SDW water plus kanamycin control. For egg assays, an aqueous suspension of eggs at various developmental stages, including 35 eggs that each contained either a J1 or a J2, was prepared in 10 µl SDW water and pipetted into each well. This was followed by 190 µl extract or control, for a total of 200 µl per well. For assays with previously hatched J2, each well received approximately 35 J2 in 10 µl SDW, and then 190 µl of extract (200 µl total per well). The microwell plates were covered by plastic adhesive sealing film (Excel Scientific, Inc., Victorville CA), the lids placed onto the plates and sealed with Parafilm (Bemis, Neenah, WI), and the nematodes incubated at 26°C. For assays with immersed J2, active J2 (showing any movement within 5 sec) and inactive J2 (no movement after 5 sec) were counted on days 1 and 2. The J2 were then rinsed twice with SDW, incubated in the second rinse, and active vs. inactive J2 counted the next day (day 3 rinsed). Inactive J2 after rinsing were considered nonviable. For the egg bioassays, total hatched J2, and active/inactive J2, were counted on days 2, 5, and 7.

### Statistical analyses

Data were analyzed with the statistical package JMP 14.2.0 (SAS Institute, Cary, NC). Differences among treatments were determined by ANOVA, and for normally distributed data, means were compared using Tukey Kramer’s adjustment for multiple comparisons (*P* ≤ 0.05). For nonparametric data, a Kruskal–Wallis test with a Wilcoxon test was used for each pair of multiple comparisons (*P* ≤ 0.05). The analyses used are indicated in the footnote of each table. Results from assays of eggs and J2 in extracts were analyzed for the highest concentrations: 200 and 400 µg/ml.

## Results

### Loline analysis

Both E+ and E− plants from all five of the *Festulolium* lines were tested for *N*-formylloline (NFL), *N*-acetylloline (NAL), *N*-acetylnorloline (NANL), and *N*-methylloline (NML). Endophyte-free plants did not have lolines in the shoots or roots. Shoots from E+ lines contained all four lolines, with NFL being found in the highest amounts (Table 2). Total lolines were higher in shoots than in roots. Roots from E+ lines contained NFL. In addition, NAL was isolated from U8 E+ and U10 E+ roots, and NANL from U8 E+ roots. When U2 E+ and U5 E+ plants were inoculated with *M. incognita*, total lolines were lower in the shoots. Roots contained NFL, as in uninoculated plants, and small amounts of NAL, NANL, and NML.

**Table 2.** Loline alkaloid concentrations µg/g cubic decimeter (DM) in shoots and roots of five *Festulolium* lines colonized by *Epichloë uncinata* (E+).

	Shoots					Roots				
*Festulolium* line, endophyte, RKN^a^ status	NFL^b^	NAL	NANL	NML	Total	NFL	NAL	NANL	NML	Total
*FHCF0802, U2 E+*										
−RKN	5,300	1,635	1,040	428	8,403	358	0	0	0	358
+RKN	577	248	150	56	1,032	214	61	38	9	323
*FHAC0802, U5 E+*										
−RKN	5,300	1,641	1,506	513	8,960	270	0	0	0	270
+RKN	1,294	418	541	210	2,462	415	129	89	54	687
*FHCD0802, U6 E+*										
−RKN	4,595	949	787	679	9,121	596	0	0	0	596
*FHAB0802, U8 E+*										
−RKN	3,419	931	689	379	5,417	551	25	14	0	590
*FHCD0802, U10 E+*										
−RKN	3,551	853	652	397	5,453	531	17	0	0	548

**Notes:** *Festulolium* lines with the U2 and U5 endophyte strains were also tested after inoculation with the root-knot nematode (RKN) *Meloidogyne incognita*. a−RKN = not inoculated with *M. incognita*; +RKN = inoculated with *M. incognita*; bNFL = N-formylloline; NAL = N-acetylloline; NANL = N-acetylnorloline; NML = N-methylloline. Total loline = NFL + NAL + NANL + NML.

### Eggs immersed in methanolic extracts from U6 E+ and U6 E− roots and shoots

For the analyzed rates of the extracts, hatch was not significantly lower in any extract treatment than in the controls on any day (data not shown). The percentage of active J2 that hatched from the eggs was not significantly decreased by any treatment on day 2, nor by any root extracts on day 5 (data not shown). However, 200 µg/ml U6 E+ and U6 E− shoot extracts and 400 µg/ml U6 E− shoot extracts significantly reduced % active J2 on day 5 compared with the corresponding 0.5 or 1.0% DMSO controls (Table 3). The differences were small, ranging from 6.1 to 8.1% decreases in J2 activity. By day 7, most of the root extracts (except U6 E+ at 200 µg/ml) significantly decreased % active J2 (12.7-18.3%) compared with the corresponding controls. All shoot extracts resulted in significant decreases in J2 activity on day 7. None of the decreases were large (9.0-17.2%). The extracts did not differ significantly from each other in efficacy on any day, regardless of the presence or absence of endophyte in the plant.

**Table 3.** *Meloidogyne incognita* egg hatch and second-stage juvenile (J2) activity in methanolic extracts from roots and shoots of *Festulolium* lines FHCD0802 BUS 10-12 U6 E+ and FHCD0802 U6 E−.

	Day 5	Day 7	
	% active	% active	
% DMSO in controls or µg/ml extract	Shoots^a^	Roots	Shoots
Water	97.1 a	94.3 a	94.3 a
Water + K^b^	94.4 abc	94.5 a	94.5 a
0.5% DMSO	95.5 ab	93.7 ab	93.7 a
1.0% DMSO	94.7 ab	95.5 a	95.5 a
U6 E+ 200 µg/ml	87.8 cd	84.9 bc	85.3 b
	(8.1%)^c^	–	(9.0%)
U6 E− 200 µg/ml	89.7 d	81.8 c	83.6 b
	(6.1%)	(12.7%)	(10.8%)
U6 E+ 400 µg/ml	90.8 bcd	83.3 c	79.1 b
	–	(12.8%)	(17.2%)
U6 E− 400 µg/ml	88.8 cd	78.0 c	83.6 b
	(6.2%)	(18.3%)	(12.5%)

**Notes:** Eggs were immersed in the extracts. aFor day 5, means within a column followed by the same letter are not significantly different according to a Kruskal–Wallis test with a Wilcoxon test for each pair of multiple comparisons (*P* ≤ 0.05). For day 7, means within a column followed by the same letter are not significantly different according to Tukey’s adjustment for multiple comparisons (*P* ≤ 0.05); bWater + K = water plus kanamycin monosulfate, which was added to all treatments except the water control; cNumbers in parentheses are percentage decreases in treatments that significantly reduced % active J2 compared with the corresponding controls: the 0.5% DMSO control for 200 ug/ml, and the 1.0% DMSO control for 400 ug/ml.

### Previously hatched J2 immersed in methanolic extracts from U6 E+ and U6 E− roots and shoots

On day 1, the only treatment at the 200 µg/ml and 400 µg/ml concentrations causing a significant loss in J2 activity was root extract from U6 E− 200 µg /ml (10.3% decrease compared with 0.5% DMSO; Table 4). This extract was more active than U6 E+ or U6 E− 400 µg/ml extracts. No shoot extract affected % active J2 on day 1. On day 2, no root extracts significantly affected % J2 activity compared with the DMSO controls or each other. Shoot extract from U6 E− 200 µg/ml was the only treatment that resulted in fewer active J2, reducing % J2 activity by 10.8% compared with the 0.5% DMSO treatment. However, there were no differences in activity among the shoot extract treatments on that day. When the J2 were rinsed and the treatments replaced with SDW (day 3 rinsed), all root and shoot extract treatments resulted in increased death of J2. Compared with the corresponding DMSO treatments, U6 E− 400 µg/ml extract from roots and shoots killed almost 1/3 of the J2. Extract effects on J2 viability were not significantly different from each other in roots or in shoots.

**Table 4.** *Meloidogyne incognita* second-stage juvenile (J2) activity and viability in methanolic extracts from roots and shoots of *Festulolium* lines FHCD0802 BUS 10-12 U6+ and FHCD0802 U6−.

	Day 1, % active J2		Day 2, % active J2		Day 3 rinsed, % viable J2	
% DMSO in controls or µg/ml extract	Roots^a^	Shoots	Roots	Shoots	Roots	Shoots
Water	89.2 a	89.2 ab	91.4 a	91.4 a	86.7 a	86.7 a
Water + K^b^	86.2 ab	86.2 ab	91.4 ab	91.4 ab	88.4 a	88.4 a
0.5% DMSO	87.7 a	87.7 ab	90.5 ab	90.5 a	85.8 a	85.8 a
1.0% DMSO	88.3 a	88.3 ab	87.2 ab	87.2 abc	85.4 a	85.4 a
U6 E+ 200 µg/ml	82.8 ab	85.4 b	86.1 ab	88.8 abc	70.0 b	68.7 b
	–	–	–	–	(18.4%)	(19.9%)
U6 E− 200 µg/ml	78.7 b	84.4 b	84.0 ab	80.7 bc	68.9 b	66.2 b
	(10.3%)^c^	–	–	(10.8%)	(19.7%)	(22.8%)
U6 E+ 400 µg/ml	90.4 a	90.9 ab	80.9 b	81.6 bc	70.0 b	71.2 b
	–	–	–	–	(18.0%)	(16.6%)
U6 E− 400 µg/ml	89.0 a	94.6 a	81.3 b	80.2 c	61.2 b	60.7 b
	–	–	–	–	(28.3%)	(28.9%)

**Notes:** Previously hatched J2 were immersed in the extracts. ^a^Means within a column followed by the same letter are not significantly different according to Tukey’s adjustment for multiple comparisons (*P* ≤ 0.05); ^b^Water + K = water plus kanamycin monosulfate, which was added to all treatments except the water control; ^c^Numbers in parentheses are percentage decreases in treatments that significantly reduced % active J2 compared with the corresponding controls: the 0.5% DMSO control for 200 ug/ml, and the 1.0% DMSO control for 400 ug/ml.

### *Festulolium* inoculated with root-knot nematode (RKN) in the greenhouse

*Festulolium* lines ±U2 and ±U5 endophytes were tested in the same trials, and lines ±U6, ±U8 and ±U10 endophytes in another set of trials. In the former trials, U2 E−, U2 E+, U5 E− and U5 E+ plants had similar shoot fresh weights in Trial 1, while U2 E+ plants had smaller shoots than the other lines in Trial 2 (Table 5). Root fresh weights were not significantly different among the *Festulolium* lines in either trial. The susceptible pepper, which was included for comparing nematode populations on a known host plant, had smaller roots than the *Festulolium* plants. Root gall indices were 0 on all *Festulolium* lines in Trial 1 and low in Trial 2, while pepper had high root gall indices in both trials. Neither total numbers of eggs nor eggs per g of root were significantly different among *Festulolium* lines in either trial, nor did they differ significantly among plants with and without endophytes. No eggs were observed from roots of U2 E+ plants in Trial 2. Egg numbers on all *Festulolium* plants were much lower than those recorded from pepper plants.

**Table 5.** Plant vigor and *Meloidogyne incognita* population densities from *Festulolium* lines, plus and minus the endophytes U2 and U5, and from susceptible pepper plants in the greenhouse.

	Shoot fresh weight^a^ (g)		Root Fresh weight (g)		Root gall index^b^		Total eggs per root system		Eggs per g of root	
Plant and endophyte	Trial 1	Trial 2	Trial 1	Trial 2	Trial 1	Trial 2	Trial 1	Trial 2	Trial 1	Trial 2
FHCF0802 U2 E−	33.0 a	71.4 a	98.1 aA	66.2 aAB	0	0.7 aB	817 aB	1,467 aB	9.7 aB	21.3 aB
FHCF0802 U2 E+	31.4 a	50.9 b	86.1 aAB	61.3 aB	0	1.0 aB	700 aB	0 aB	7.8 aB	0.0 aB
FHAC0802 U5 E−	29.3 a	70.3 a	41.4 aBC	63.3 aB	0	0.3 aB	817 aB	800 aB	19.5 aB	13.1 aB
FHAC0802 U5 E+	33.4 a	73.1 a	49.3 aABC	99.9 aA	0	0.3 aB	933 aB	1,733 aB	21.8 aB	16.1 aB
Pepper	NA	NA	14.1 C	15.7 C	25	25.0 A	225,108 A	104,000 A	15,975.0 A	6,239.7 A

**Notes:** aFor shoot fresh weight, root fresh weight, root gall index Trial 2 without pepper, total eggs per root system, and eggs per g of root Trial 1 and Trial 2 without pepper, means within a column followed by the same letter are not significantly different according to Tukey’s adjustment for multiple comparisons (*P* ≤ 0.05). For root gall index Trial 2 with pepper, and eggs per g of root Trial 2 with pepper, means within a column followed by the same letter are not significantly different according to a Kruskal–Wallis test with a Wilcoxon test for each pair of multiple comparisons (*P* ≤ 0.05). Lower case letters are for comparisons among *Festulolium* plants only. Upper case letters are for comparison among all plants, including pepper; broot gall indices follow Daulton and Nusbaum (1961). 0 = no galls, 1 = 1 to 4 galls, 5 = 5 to 25 galls, 10 = 26 to 100 galls and 25 = more than 100 galls.

In trials with *Festulolium* lines ±U6, ±U8, and ±U10, there was a tendency for U10 E+ plants to have the lowest fresh shoot weights, but the roots were not significantly smaller than most other lines (Table 6). Root fresh weights did not significantly differ among *Festulolium* lines in Trial 1, while U10 E− plants had the largest shoots and roots in Trial 2. Pepper root weights were similar to most *Festulolium* root weights. In Trial 1, U8 E− plants had a higher mean root gall index than the other *Festulolium* associations when pepper was included in the analysis. With that exception, root gall indices, total eggs per root system, and eggs per g of root did not differ significantly among *Festulolium* lines or with endophyte status, but all were lower than numbers recorded from pepper. No galls were observed on U8 E− plants in Trial 2. No eggs were found in the root systems of U6 E−, U6 E+, or U8 E− plants in Trial 2.

**Table 6.** Plant vigor and *Meloidogyne incognita* population densities from *Festulolium* lines, plus and minus the endophytes U6, U8, and U10, and from susceptible pepper plants in the greenhouse.

	Shoot fresh weight^a^ (g)		Root fresh weight (g)		Root gall index^b^		Total eggs per root system		Eggs per g of root	
Plant and endophyte	Trial 1	Trial 2	Trial 1	Trial 2	Trial 1	Trial 2	Trial 1	Trial 2	Trial 1	Trial 2
FHCD0802 U6 E−	24.0 a	43.2 ab	16.1 aAB	34.7 abAB	6.7 aB	8.3 aB	10,533 aB	0 aB	500.0 aB	0.0 aB
FHCD0802 U6 E+	17.1 ab	33.9 ab	12.2 aAB	24.7 bBC	6.7 aB	5.0 aB	3,067 aB	0 aB	284.5 aB	0.0 aB
FHAB0802 U8 E−	19.9 ab	31.7 b	14.5 aAB	25.0 abBC	8.3 aA	0.0 aB	933 aB	0 aB	63.1 aB	0.0 aB
FHAB0802 U8 E+	11.7 ab	29.2 b	13.1 aAB	17.4 bBC	3.3 aB	6.7 aB	933 aB	53 aB	71.2 aB	3.0 aB
FHCD0802 U10 E−	18.1 ab	51.5 a	14.0 aAB	47.3 aA	6.7 aB	6.7 aB	1,333 aB	40 aB	95.3 aB	1.0 aB
FHCD0802 U10 E+	6.5 b	29.7 b	4.0 aB	22.2 bBC	5.3 aB	1.7 aB	533 aB	13 aB	803.2 aB	0.7 aB
Pepper	NA	NA	17.0 A	13.5 C	25.0 A	25.0 A	466,933 A	119,333 A	27,644.3 A	8,552.8 A

**Notes:** aFor shoot fresh weight, root fresh weight, root gall index Trial 2, and eggs per g of root Trial 1 with pepper, means within a column followed by the same letter are not significantly different according to Tukey’s adjustment for multiple comparisons (*P* ≤ 0.05). For root gall index Trial 1, total eggs per root system, and eggs per g of root Trial 1 without pepper and Trial 2 with and without pepper, means within a column followed by the same letter are not significantly different according to a Kruskal–Wallis test with a Wilcoxon test for each pair of multiple comparisons (*P* ≤ 0.05). Lower case letters are for comparisons among *Festulolium* plants only. Upper case letters are for comparison among all plants, including pepper; broot gall indices follow Daulton and Nusbaum (1961). 0 = no galls, 1 = 1 to 4 galls, 5 = 5 to 25 galls, 10 = 26 to 100 galls and 25 = more than 100 galls.

Following the first harvest, pepper seedlings were transplanted into pots that had contained plants and into *M. incognita*-inoculated fallow pots. In pots from which *Festulolium* U2 E+, U2 E−, U5 E+ and U5 E− plants had been harvested, pepper root fresh weights in both trials were generally similar on plants following all *Festulolium* lines (Table 7). The root gall indices tended to be lowest on pepper following U5 E+ and fallow soil pots. Total eggs per root system and eggs per g of root were lowest in both trials from pepper planted into fallow pots. Total eggs and eggs per g of root did not differ among the other treatments in Trial 1, but in Trial 2 the numbers were highest on pepper, which was significantly different from all treatments except U2 E− *Festulolium* plants.

**Table 7.** *Meloidogyne incognita* population densities on pepper in soil that was previously planted to *Festulolium* with or without a U2 or U5 endophyte, to susceptible pepper, or left fallow in the greenhouse.

	Root fresh weight^a^ (g)		Root gall index^b^		Total eggs per root system		Eggs per g of root	
Previous treatment: Fallow, *Festulolium* line and endophyte, or pepper	Trial 1	Trial 2	Trial 1	Trial 2	Trial 1	Trial 2	Trial 1	Trial 2
Fallow	6.8 ab	20.3 ab	4.3 b	0.5 c	4,550 b	200 c	679.8 b	9.2 c
FHCF0802 U2 E−	5.2 b	22.5 ab	22.5 a	25.0 ab	58,450 a	135,200 ab	10,316.0 a	5,047.7 ab
FHCF0802 U2 E+	6.7 ab	23.5 ab	17.5 ab	11.7 bc	37,625 a	10,267 bc	5,382.0 a	613.0 bc
FHAC0802 U5 E−	6.4 ab	19.5 ab	13.3 ab	18.3 ab	34,650 a	18,267 b	5,264.2 a	823.7 b
FHAC0802 U5 E+	7.3 a	25.4 a	7.2 b	3.3 c	21,700 ab	533 bc	3,045.7 ab	18.3 bc
Pepper	2.6 c	11.7 b	16.0 ab	25.0 a	17,010 a	207,467 a	6,293.4 a	16,420.5 a

**Notes:** aFor root fresh weight and root gall index, means within a column followed by the same letter are not significantly different according to Tukey’s adjustment for multiple comparisons (*P* ≤ 0.05). For total eggs per root system and eggs per g of root, means within a column followed by the same letter are not significantly different according to a Kruskal–Wallis test with a Wilcoxon test for each pair of multiple comparisons (*P *≤ 0.05); broot gall indices follow Daulton and Nusbaum (1961). 0 = no galls, 1 = 1 to 4 galls, 5 = 5 to 25 galls, 10 = 26 to 100 galls, and 25 = more than 100 galls.

Pepper seedlings were also transplanted after harvest of pepper and *Festulolium* U6 E+, U6 E−, U8 E+, U8 E−, U10 E+ and U10 E− plants. In those trials, the pepper root fresh weights were overall lowest on pepper following pepper (Table 8). Root gall indices were low on pepper transplanted into fallow pots in both trials. In Trial 1, root gall indices were similar to each other among all other treatments. However, in Trial 2, root galls were not evident on pepper plants following U8 E−, U8 E+, or U10 E+ plants. Total eggs per root system and eggs per g of root were lowest on pepper planted into fallow pots in Trial 1. In Trial 2, these numbers were highest on pepper following pepper, and not significantly different among pepper plants following any *Festulolium* treatment. Total eggs and eggs per g of root on pepper following U8 E+ and U10 E+ were significantly lower than numbers on pepper planted into fallow soil.

**Table 8.** *Meloidogyne incognita* population densities on pepper in soil that was previously planted to *Festulolium* with or without a U6, U8, or U10 endophyte, to pepper, or left fallow in the greenhouse.

	Root fresh weight^a^ (g)		Root gall index^b^		Total eggs per root system		Eggs per g of root	
Previous treatment: Fallow, *Festulolium* line and endophyte, or pepper	Trial 1	Trial 2	Trial 1	Trial 2	Trial 1	Trial 2	Trial 1	Trial 2
Fallow	6.4 a	7.8 bc	8.3 b	0.8 c	3 c	3,233 c	0.5 b	533.5 b
FHCD0802 U6 E−	6.3 a	14.1 a	20.0 a	18.3 ab	193,600 ab	1,333 bcd	33,590.0 a	81.0 bc
FHCD0802 U6 E+	6.1 a	11.8 ab	25.0 a	15.0 ab	403,467 a	12,000 bd	56,056.7 a	1,105.0 bc
FHAB0802 U8 E−	5.3 a	10.1 abc	25.0 a	0.0 bc	87,200 ab	533 d	21,668.7 a	73.0 bc
FHAB0802 U8 E+	5.6 a	10.6 abc	25.0 a	0.0 bc	37,333 ab	1,333 d	6,400.0 a	123.0 c
FHCD0802 U10 E−	5.8 a	10.5 abc	25.0 a	18.3 ab	47,200 ab	5,067 bcd	8,137.7 a	485.3 bc
FHCD0802 U10 E+	6.6 a	11.1 ab	25.0 a	0.0 bc	35,733 ab	800 d	5,269.7 a	78.7 c
Pepper	2.4 b	5.5 c	25.0 a	25.0 a	10,800 b	100,033 a	5,301.5 a	17,842.3 a

**Notes:** aFor root fresh weight, means within a column followed by the same letter are not significantly different according to Tukey’s adjustment for multiple comparisons (*P* ≤ 0.05). For root gall index, total eggs per root system, and eggs per g of root, means within a column followed by the same letter are not significantly different according to a Kruskal–Wallis test with a Wilcoxon test for each pair of multiple comparisons (*P* ≤ 0.05); broot gall indices follow Daulton and Nusbaum (1961). 0 = no galls, 1 = 1 to 4 galls, 5 = 5 to 25 galls, 10 = 26 to 100 galls, and 25 = more than 100 galls.

## Discussion

In this study, the presence or absence of an *E. uncinata* endophyte did not change activity of methanolic extracts from *Festulolium* against *M. incognita* in laboratory assays. It was also demonstrated that inoculation of *Festulolium* E+ plants with *M. incognita* resulted in an altered loline profile compared with uninoculated plants. In greenhouse trials, host status to *M. incognita* was not affected by *Festulolium* line or endophyte status.

Shoots of all five *Festulolium* E+ associations contained all four types of lolines (NFL, NAL, NANL, and NML). NFL was also found in all roots, and small amounts of other lolines in two *Festulolium*/endophyte associations. This is similar to previously reported loline analyses from *Festulolium* (Barker et al., 2015b).

In the current study, root loline profiles changed when plants (U2 E+ and U5 E+) were inoculated with *M. incognita*. All four types of lolines were present in the roots inoculated with the nematode. Total loline concentrations in *Festulolium* U5 E+ roots were higher in inoculated plants than in uninoculated plants. However, no such increase was observed in U2 E+ roots from inoculated plants.

In shoots, total loline concentrations were ca. 3.5× (U5 E+ plants) to 8× (U2 E+ plants) higher in plants without *M. incognita* than in plants inoculated with nematodes. Patchett et al. (2008) observed that loline concentrations were lower in crowns of meadow fescue when the plants were attacked by grass grubs (*Costelytra zealandica*), but total loline concentrations in shoots were not different with or without grass grubs. We did not analyze crowns for loline content, but the decrease in total lolines in shoots of *Festulolium* plants inoculated with *M. incognita* correlates with the observation that translocation of lolines from other areas of the plant may be involved in changes in root lolines (Patchett et al., 2008).

Methanolic extracts from greenhouse-grown *Festulolium* line FHCD0802 did not inhibit *M. incognita* egg hatch, with or without the presence of the U6 endophyte in the plant. However, root and shoot extracts were lethal to J2. *Meloidogyne incognita* J2 death was not higher in the E+ root and shoot extracts than in the E− extracts, despite the presence of lolines in E+ plants. By comparison, methanolic extracts from roots of tall fescue (*Schedonorus arundinaceus* (Schreb.) Dumort = *Festuca arundinacea Schreb*.) plants associated with an *E. coenophiala* endophyte were repellent to *P. scribneri* (after the plants had been growing for at least 45 d), and were nematostatic; extracts from non-infected plant roots were attractants and not active against the nematodes, indicating differences in root metabolites (Bacetty, Snook, Glenn, Noe, Hill, Culbreath, Timper, Nagabhyru and Bacon, 2009; Bacetty, Snook, Glenn, Noe, Nagabhyru and Bacon, 2009). The loline alkaloid NFL was a weak repellent to *P. scribneri* at 50 to 200 µg/ml, an attractant at 1 to 20 µg/ml, and not lethal at any tested concentration (Bacetty, Snook, Glenn, Noe, Nagabhyru and Bacon, 2009).

Unlike the studies with tall fescue and *P. scribneri*, metabolites toxic to *M. incognita* were produced by *Festulolium* regardless of endophyte status. This indicates that nematotoxicity of *Festulolium* extracts was likely caused by active metabolites other than lolines. Activity of plant compounds against other organisms may be a result of one major compound or a combination of diverse natural products. As just one example of potentially active natural products, phenolic acids are found in all plants, and some are nematicidal (López-Martínez et al., 2011). Phenols in root exudates can vary with endophyte status and cultivar (Guo et al., 2015). Despite the possibility for such differences in *Festulolium* as well, effects of extracts from U6 E+ and U6 E− plants were not significantly different, and the active metabolite(s) were not identified at this time.

Greenhouse studies with all five *Festulolium* lines, with and without endophytes, indicated that all were poor hosts for *M. incognita*. *Festulolium* line FHCF0802 ± U2 was compared with FHAC0802 ± U5, whereas lines FHCD0802 ± U6, FHAB0802 ± U8, and FHCD0802 ± U10 were compared with each other. Host status was not affected by *Festulolium* line nor by endophyte presence or absence. Nematode/*Festulolium* interactions have not been widely studied, but these results differ from those reporting that endophyte status does affect insect feeding. The New Zealand weta (cricket; *Hemiandrus* sp. ‘promontorius’) preferred feeding on endophyte-free *Festulolium loliaceum* and *Lolium perenne*, rather than *F. loliaceum* associated with *E. uncinata* (producing loline alkaloids) or *Festuca rubra* associated with *Epichloë festucae* (which produces ergovaline and Lolitreme B) (Nboyine et al., 2017). Similarly, common true katydids (*Pterophylla camellifolia*), fall armyworms (*Spodoptera frugiperda*), and Bird cherry-oat aphids (*Rhopalosiphum padi*) preferred to consume *Festuca subverticillata* (nodding fescue) plants without endophyte in preference to plants with endophyte (Afkhami and Rudgers, 2009). Conversely, while dusky and eastern lubber grasshoppers (*Encoptolophus costalis* and *Romalea microptera*, respectively) also showed a preference, they consumed more of the endophyte-associated plants (Afkhami and Rudgers, 2009).

Effects of endophyte-grass cultivar combinations on nematode infection have primarily been studied with tall fescue and species of *Meloidogyne* or *Pratylenchus*. Results are variable, as indicated for *Meloidogyne* species on tall fescue and Italian ryegrass with varying endophytes (Table 9). For example, *M. arenaria* infected tall fescue and Italian ryegrass regardless of cultivar or endophyte status. In addition, several tall fescue cultivars were poor or nonhosts for *M. incognita*; endophyte status was not a factor. This is similar to our results with *Festulolium*, which was a poor host regardless of line or endophyte presence under the conditions of this study. Italian ryegrass cultivars were hosts for *M. incognita*, notwithstanding cultivar/endophyte association. However, ‘Kentucky 31’ E+ and E− were both hosts, indicating that cultivar was more important than endophyte association. Tall fescue ‘Bulldog 51’ with a toxic endophyte was a host for *M. javanica*, but ‘Jesup Max-Q’ with a nonergot-alkaloid producing endophyte was not. This result is comparable to the finding that production of ergot alkaloids did not affect *Pratylenchus scribneri* populations on perennial ryegrass (Panaccione et al., 2006). For *M. marylandi*, host status of tall fescue ‘Kentucky 31’ and ‘Genotype GA 1987’ varied with endophyte, while all other Genotype GA/endophyte associations were poor hosts. These results as a whole indicate that susceptibility to nematodes is unpredictable, and grass line/fungal endophyte associations must be tested individually for susceptibility to each nematode species.

**Table 9.** Host status of grass cultivar/endophyte associations to *Meloidogyne* spp. Grasses: tall fescue (*Schedonorus arundinaceus*); Italian ryegrass (*Festuca perennis*; syn. *Lolium multiflorum*).

Tall Fescue Cultivar (Endophyte)	*M. arenaria* Peanut root-knot nematode	*M. hapla* Northern root-knot nematode	*M. incognita* Southern root-knot nematode	*M. javanica* Javanese root-knot nematode	*M. marylandi* Maryland root-knot nematode
Bulldog 51 (E+ toxic)^a^	Host^b^	–	Poor Host^b^	Host^b^	–
Jesup (Wild Type) (E+ toxic)	–	–	Nonhost^b^	–	–
Jesup (Max-Q) (E+ nontoxic AR542)	Host^b^	Nonhost^b^	Nonhost^b^	Poor Host^b^	–
Jesup (E− no endophyte)	–	–	Nonhost^b^	–	–
Georgia 5 (E+ toxic)	–	–	Nonhost^b^	–	–
Kentucky 31 (E+)	–	–	Host^c^	–	Poor Host^d^
Kentucky 31 (E− no endophyte)	–	–	Host^c^	–	Host^d^
Genotype GA 1987 (E+)	–	–	–	–	Host^e^
Genotype GA 1987 (E−)	–	–	–	–	Poor host^e^
Genotype GA 2109 (E+)	–	–	–	–	Poor host^e^
Genotype GA 2109 (E−)	–	–	–	–	Poor host^e^
Genotype GA 2125 (E+)	–	–	–	–	Poor host^e^
Genotype GA 2125 (E−)	–	–	–	–	Poor host^e^
Genotype GA 3084 (E+)	–	–	–	–	Poor host^e^
Genotype GA 3084 (E−)	–	–	–	–	Poor host^e^
Genotype GA87-122 (E+)	–	–	–	–	Poor host^f^
Genotype GA87-122 (E−)	–	–	–	–	Host^f^
*Italian ryegrass Cultivar (Endophyte)*					
Bishamon (E+)	Host^g^	–	Host^g^	–	–
Bishamon (E−)	Host^g^	–	Host^g^	–	–
JFIR-18 (E+)	Host^g^	–	Host^g^	–	–
JFIR-18 (E−)	Host^g^	–	Host^g^	–	–

**Notes:** aDescribed as toxic (= toxic to mammals); endophyte produces ergot alkaloids. Nontoxic=endophyte does not produce ergot alkaloids; bNyczepir and Meyer (2010); cJia et al. (2013); dKimmons et al. (1990); eKirkpatrick et al. (1990). Although listed in the paper as *M. graminis*, the nematode was likely *M. marylandi* (personal communication, T. Kirkpatrick, 2019); fElmi et al. (2000); gUesugi et al. (2014).

Although the *Festulolium* plants in our study were poor hosts for *M. incognita*, nematode populations were sometimes higher on pepper plants following *Festulolium* than on pepper following fallow soil. The nematodes available to attack the pepper seedlings would have included the original surviving inoculum, eggs dislodged from the previous pepper or *Festulolium* roots, and J2 that hatched before the plants were removed from the soil. It is likely that compared with fallow soil, enough inoculum was generated on some *Festulolium* plants to increase the infection rate on the following crop plant.

In summary, these studies indicate that the tested *Festulolium* lines, with and without endophytes, were poor hosts for *M. incognita*. Assays with one line demonstrated that *Festulolium* ± a colonizing endophyte can produce compounds lethal to *M. incognita* J2. Field studies would indicate whether planting these *Festulolium* lines would contribute to suppression of plant-parasitic nematodes in pastures.
